# Direct comparison of multiple computer-aided polyp detection systems

**DOI:** 10.1055/a-2147-0571

**Published:** 2023-10-05

**Authors:** Joel Troya, Boban Sudarevic, Adrian Krenzer, Michael Banck, Markus Brand, Benjamin M. Walter, Frank Puppe, Wolfram G. Zoller, Alexander Meining, Alexander Hann

**Affiliations:** 1 Interventional and Experimental Endoscopy (InExEn), Department of Internal Medicine II, University Hospital Würzburg, Würzburg, Germany; 2 Bavarian Cancer Research Center, Würzburg, Germany; 3 Department of Internal Medicine and Gastroenterology, Katharinenhospital, Stuttgart, Germany; 4 Artificial Intelligence and Knowledge Systems, Institute for Computer Science, Julius-Maximilians-Universität, Würzburg, Germany; 5 Department of Internal Medicine I, University Hospital Ulm, Ulm, Germany

## Abstract

**Background and study aims** Artificial intelligence (AI)-based systems for computer-aided detection (CADe) of polyps receive regular updates and occasionally offer customizable detection thresholds, both of which impact their performance, but little is known about these effects. This study aimed to compare the performance of different CADe systems on the same benchmark dataset.

**Methods** 101 colonoscopy videos were used as benchmark. Each video frame with a visible polyp was manually annotated with bounding boxes, resulting in 129 705 polyp images. The videos were then analyzed by three different CADe systems, representing five conditions: two versions of GI Genius, Endo-AID with detection Types A and B, and EndoMind, a freely available system. Evaluation included an analysis of sensitivity and false-positive rate, among other metrics.

**Results** Endo-AID detection Type A, the earlier version of GI Genius, and EndoMind detected all 93 polyps. Both the later version of GI Genius and Endo-AID Type B missed 1 polyp. The mean per-frame sensitivities were 50.63 % and 67.85 %, respectively, for the earlier and later versions of GI Genius, 65.60 % and 52.95 %, respectively, for Endo-AID Types A and B, and 60.22 % for EndoMind.

**Conclusions** This study compares the performance of different CADe systems, different updates, and different configuration modes. This might help clinicians to select the most appropriate system for their specific needs.

## Graphical abstract

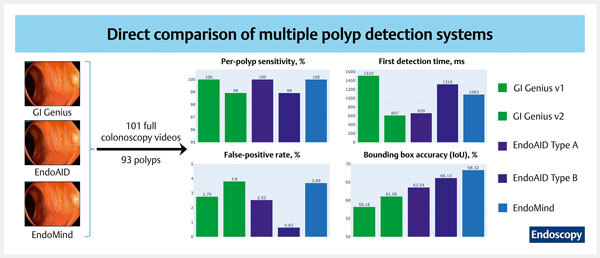

## Introduction

Screening colonoscopy is considered to be an effective prevention measure to decrease colorectal cancer. However, it has been reported that 17 %–48 % of adenomas are missed during this procedure 1 2 . Systems for computer-aided detection (CADe) of polyps are intended to work as an adjunct to the endoscopist, helping them to identify polyps. The first CADe system approved in Europe for commercial distribution was GI Genius (Medtronic, Ireland), in 2019 3 . Since then, there has been growing interest in demonstrating the efficiency of such devices. Prospective randomized controlled studies show an increase in the adenoma detection rate when endoscopists use CADe systems 4 5 6 7 8 9 10 11 12 13 .

CADe systems are developed by training neural networks, usually with previously annotated images. A properly trained model can then produce an output using new data. However, the output cannot be predicted in all possible scenarios. It is essential to know the nature of the training data to know what a CADe system is capable of. Unfortunately, CADe manufacturers do not provide any information about this and/or about how the system’s algorithm was developed. In addition, each CADe system has been validated with different data, hindering comparison of their performance. Furthermore, updates to CADe software affect their performance. For all these reasons, it is necessary to continuously undertake studies in which the performances of systems and updates are compared using the same data.

This study includes the performance of two versions of GI Genius, an early version (software current in March 2020, version 1.0), from now on called “first version,” and a subsequent version (software current in October 2021, version 2.0.1), from now on called “second version.” It also includes the performance of Endo-AID (Olympus Medical Systems, Japan; software current in March 2022) in both of its detection Types A and B, and finally the freely available system, EndoMind. Our aim is to compare the sensitivity of the systems, using a fully annotated dataset to characterize their detection strength. Other metrics are compared such as false-positive rate and “first detection time” (FDT), a measure of how quickly the CADe system detects a polyp. In addition, “intersection over union” (IoU) is calculated, an evaluation of the system’s ability to accurately locate polyps.

## Methods

### Study design

#### Ethical considerations

Details of ethics committee approval can be found in the Supplementary material (available online-only).

#### Dataset

A total of 244 colonoscopy videos from different patients were recorded in the University Hospitals Würzburg and Ulm (Germany). The videos were recorded between March 2019 and April 2020 in high definition video signal from the endoscopy processor (Olympus CV-190; Olympus).

**Fig. 1:**
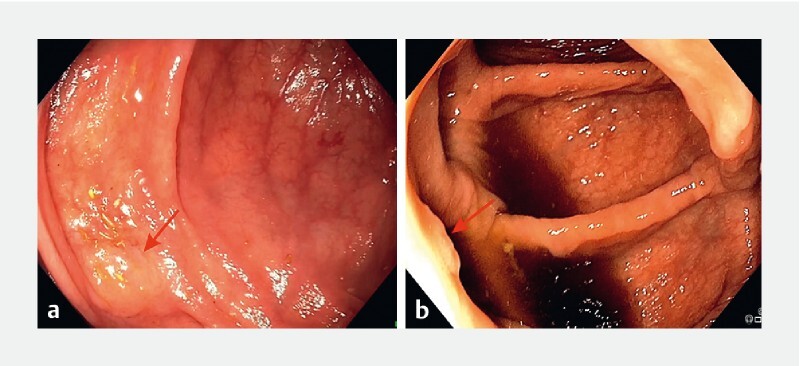
Images of polyps that were not detected: **a** by GI Genius version 2; or **b** by Endo-AID using detection Type B.

The inclusion criterion was examination carried out for screening purposes or post-polypectomy surveillance. Exclusion criteria are described in the Supplementary material (including **Fig. 1 s** ).

#### Creation of benchmark

A board-certified gastroenterologist and experienced endoscopist, with over 4000 colonoscopies performed, screened all the videos as described previously 14 . Using a custom-made annotation tool, the colonoscopies were analyzed in a deep frame-by-frame process and in each frame that contained a polyp, a bounding box was drawn around the lesion 15 . This provided the “ground-truth” dataset.

**Fig. 2:**
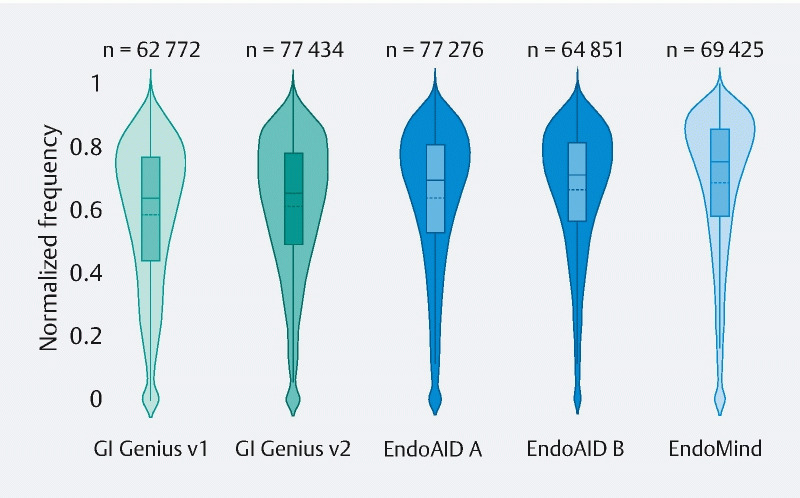
Violin plot showing the distribution of the intersection over union (IoU) values between the ground-truth manually annotated areas (as bounding boxes) identifying polyps, and the displayed computer-aided detection (CADe) areas. The violin plot is similar to a box plot, with the addition of a rotated kernel density plot on each side. Additionally, a box plot is shown: the ends of the box represent the lower and upper quartiles; the continuous line inside the box shows the median (second quartile); the dotted line represents the mean value of the IoU distribution.

Details of the benchmarking annotation process can be found in Supplementary material (including **Fig. 2 s** ).

**CADe data acquisition** All the raw colonoscopy videos were processed by each CADe system in the same way. A video converter was used to send the signal from a laptop to the CADe system (Mini Converter UpDownCross HD, Blackmagic Design, Australia). A video recorder (DeckLink Mini Recorder, Blackmagic Design) was used to record the HD signal of each CADe system. A custom algorithm to detect the bounding box locations was developed using Python (Python Software Foundation, version 3.8) (see Supplementary material).

### Outcomes

The primary outcome measure of the study was sensitivity. Per-polyp sensitivity was defined as the ratio of the total number of polyps as detected in at least one frame by the CADe, and the total number of polyps. The per-frame sensitivity was defined, for each polyp, as the ratio of the number of frames with a correctly identified image of the polyp and the total number of benchmark frames with an image of the polyp. This takes account of the duration for which polyps appear in the image frames.

**Fig. 3:**
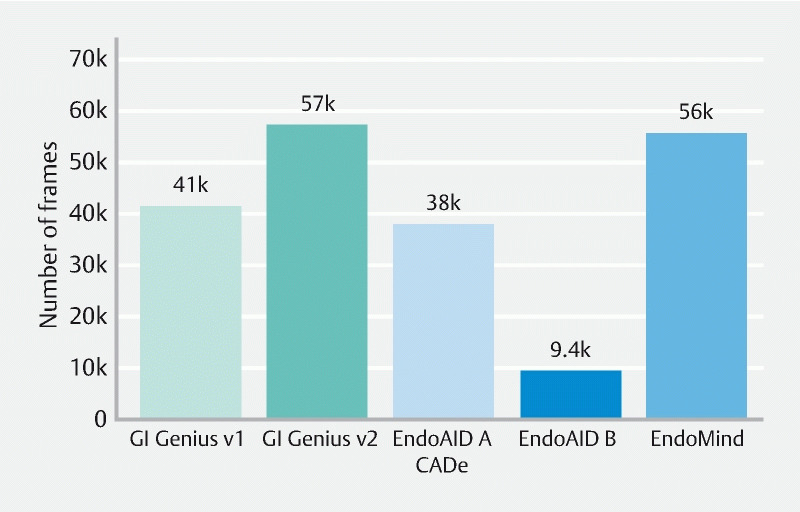
Bar plot showing the total amount of false-positive image frames triggered by each of the analyzed CADe systems.

Secondary outcomes included IoU, FDT, and false-positive rate. IoU measures the accuracy of the polyp bounding box predictions by evaluating their overlap with the ground-truth bounding box ( **Fig. 3 s** ). More details of these metrics and the statistical analysis can be found in the Supplementary material.

## Results

### Baseline characteristics

Out of 244 recorded routine colonoscopies, 143 colonoscopies met the exclusion criteria. Thus, a total of 101 colonoscopy videos were used to analyze the performance of each of the CADe systems.

From a total of 2 161 818 image frames, 464 186 were considered part of a polypectomy, 56 902 were acquired with narrow band imaging, 37 445 frames were repeated image frames due to freezes for documentation and 97 105 consisted of images of the rectum. These were all excluded.

A total of 45 (44.55 %) videos contained at least one polyp. The total number of polyps was 93 and these accounted for 129 705 (8.61 %) image frames ( **Fig. 1 s** ). In total, 1 506 180 image frames were processed by each system, resulting in a dataset of 7 530 900 images.

The patients and polyp characteristics with the accompanying histology are presented in **Table 1 s** .

### Primary outcome

#### Sensitivity

**Per-polyp sensitivity** was 100 % for the first version of GI Genius, Endo-AID in detection Type A, and EndoMind. Both GI Genius second version and Endo-AID using Type B missed 1 polyp.

GI Genius second version did not detect a sessile serrated adenoma (SSA) of type Paris 0-IIa ( Fig. 1a ) located in the right colon that was present for 10.20 seconds. This would have resulted in a 7-year delay on patient follow-up according to German and U.S. guidelines 16 17 . Endo-AID (Type B) did not detect a Paris 0-IIa polyp ( Fig. 1b ) in the right colon that was present for 0.87 seconds, and was also not detected by the endoscopist. In this case, there would not have been any delay in patient follow-up.

**Overall mean per-frame sensitivity** for each system was as follows: GI Genius first version, 50.63 % (95 %CI 45.20 %–56.07 %); GI Genius second version, 67.85 % (95 %CI 63.26 %–72.43 %); Endo-AID Type A, 65.60 % (95 %CI 60.26 %–70.95 %); Endo-AID Type B, 52.95 % (95 %CI 46.92 %–58.99 %); and EndoMind, 60.22 % (95 %CI 54.66 %–65.78 %) ( Table 1 ).

**Table 1:** Per-polyp and per-frame sensitivity for each analyzed computer-aided detection system (CADe) system.

	GI Genius version 1	GI Genius version 2	EndoAID Type A	EndoAID Type B	EndoMind
*Per-polyp sensitivity, %*	100	98.92	100	98.92	100
*Per-frame sensitivity, mean (95 % CI), %*					
Overall	50.63 (45–56)	67.85 (63–72)	65.60 (60–71)	52.95 (47–59)	60.22 (55–66)
Paris classification					
0-Ip	79.80 (56–100)	85.36 (62–100)	81.60 (59–100)	70.16 (39–100)	73.66 (39–100)
0-Is	64.96 (58–72)	79.81 (74–85)	81.44 (75–87)	72.35 (65–80)	77.37 (71–84)
0-IIa	41.52 (35–48)	60.80 (55–67)	56.88 (50–64)	42.47 (35–50)	51.10 (44–58)
Size					
< 5 mm	49.89 (43–57)	68.78 (63–74)	66.56 (60–74)	55.34 (47–63)	62.03 (54–70)
5–10 mm	58.68 (48–70)	74.93 (65–84)	74.22 (64–85)	60.54 (49–72)	67.11 (56–78)
> 10 mm	40.43 (27–54)	53.14 (40–66)	48.39 (36–61)	32.22 (19–45)	42.69 (33–52)
Location					
Right colon	47.57 (41–54)	65.21 (59–71)	63.61 (56–71)	50.31 (42–58)	57.06 (50–65)
Left colon	52.34 (42–63)	68.32 (60 – 77)	65.88 (56–75)	54.60 (44–66)	62.37 (52–72)
Rectum	57.65 (40–75)	75.73 (62–89)	71.74 (55–88)	58.69 (39–78)	66.76 (49–84)
Histology					
Hyperplastic polyp (n = 16)	58.33 (43–74)	70.46 (57–84)	64.48 (48–81)	54.24 (37–72)	61.88 (45–79)
Sessile serrated lesion (n = 18)	46.02 (32–60)	57.45 (43–72)	54.71 (39–70)	38.19 (23–54)	49.56 (36–63)
Adenoma (n = 43)	57.98 (50–66)	74.42 (68–81)	76.06 (69–83)	65.93 (58–74)	68.58 (60–77)
Other (n = 31)	37.80 (27–49)	63.12 (55–72)	56.94 (46–68)	41.63 (29–54)	53.28 (43–64)

**Median per-frame sensitivity** was significantly different between all the devices except between GI Genius second version and Endo-AID Type A ( *P* = 0.460), and GI Genius first version and Endo-AID Type B ( *P* = 0.242).

**Morphology** Across all devices the median per-frame sensitivity was significantly lower for flat polyps (51.70 %, interquartile range [IQR] 29.35 %–72.58 %) when compared to type 0-Ip (85.90 %, IQR 71.40 %–95.40 %) or type 0-Is (81.00 %, IQR 64.25 %–89.15 %).

### Secondary outcomes

#### First detection time (FDT)

**Mean FDT** for each system was as follows: GI Genius first version, 1510 ms (95 %CI 1125–1895); GI Genius second version, 607 ms (95 %CI 411–803); Endo-AID (Type A), 659 ms (95 %CI 410–909); Endo-AID (Type B), 1316 ms (95 %CI 951–1682); and EndoMind, 1083 ms (95 %CI 627–1539) ( **Table 2 s** ).

**Median FDT** was significantly different between all the systems.

**Morphology** All the systems presented a longer median FDT for polyps with 0-IIa morphology (350 ms, IQR 167–1442) when compared with 0-Ip (333 ms, IQR 133–533; *P* = 0.063) and 0-Is (233 ms, IQR 133–583; *P* = 0.002).

#### Intersection over union (IoU)

**Mean IoU** values were as follows ( Fig. 2 ): GI Genius first version, 58.18 % (95 %CI 58.0 %–58.36 %); GI Genius second version, 61.06 % (95 %CI 60.91 %–61.21 %); Endo-AID Type A, 63.54 % (95 %CI 63.38 %–63.70 %); Endo-AID Type B, 66.13 % (95 %CI 65.98 %–66.29 %); and EndoMind, 68.32 % (95 %CI 68.15 %–68.48 %).

When tested, all the mean values for IoU distribution were significantly different from one another.

#### False-positive (FP) rate ( Fig. 3 )

GI Genius first version presented a total of 41 411 FP image frames, equivalent to a rate of 2.75 % of all images. Corresponding values for GI Genius second version were 57 278 FP images (3.80 %); Endo-AID Type A, 38 012 FP images (2.52 %); EndoAid Type B, 9432 FP images (0.63 %); and the freely available EndoMind, 55 631 FP images (3.69 %).

### Results summary



**Video 1** Visualization of an adenoma as humanly detected and by computer-aided detection (CADe) systems. Top, from left to right: human box annotation, GI Genius version 1, and GI Genius version 2. Bottom, from left to right: Endo-AID Type A, Endo-AID Type B, and EndoMind.


Table 2 summarizes the above results, as well as additional metrics such as per-box mean precision for each of the systems.

**Table 2:** Summary of results of comparison of the different computer-aided detection (CADe) systems. All the metrics except per-polyp sensitivity were assessed in a frame-by-frame manner.

Metric, mean	GI Genius version 1	GI Genius version 2	EndoAID Type A	EndoAID Type B	EndoMind
Per-polyp sensitivity, %	100	98.92	100	98.92	100
Per-frame sensitivity, %	50.63	67.85	65.60	52.95	60.22
False-positive rate, %	2.75	3.80	2.52	0.63	3.69
False-positive rate per colonoscopy, %	2.43	3.40	2.41	0.51	3.90
First detection time, ms	1510	607	659	1316	1083
Intersection over union, %	58.18	61.06	63.54	66.13	68.32
Precision, %	59.66	57.01	66.58	87.20	54.99
Specificity, %	96.94	95.77	97.19	99.30	95.89
F1 score 1 , %	59.15	63.91	69.43	71.77	59.60

1 Single-value accuracy metric balancing precision (positive predictive value) and recall (sensitivity).

Video 1 shows the visualization by the different devices during the recognition of an adenoma.

## Discussion

Recently published randomized clinical trials present evidence of the ability of CADe systems such as GI Genius and Endo-AID to detect more adenomas in comparison to examinations without CADe 9 13 18 . Since then, the use of CADe systems has quickly expanded in clinical practice 11 12 19 20 . However, as already discussed, it is difficult to compare CADe systems to establish which performs better. Additionally, updates of existing systems might affect their ability to detect polyps. For these reasons, CADe systems of different manufacturers and of different versions need to be compared over time, using the same dataset under the same conditions.

In this study we compared the evolution of the GI Genius system. This has never been described before, to our knowledge. The first version might be closer to or equal to that used by Repici et al. in a study period September–November 2019 9 . The second version might resemble that also used by Repici et al. in a study period February–December 2020 18 . We have identified that the later version is significantly more sensitive than the first version and needs less time to detect polyps. However, the number of FPs is also significantly higher.

Customization of systems will be increasingly implemented 21 . In this regard, Endo-AID uses two different detection types, A and B. As described in the manual, Type A, detects more potential colorectal polyps than Type B, whereas Type B tends to suppress more false detections than Type A. Schauer et al. 22 and Gimeno-García et al., used detection Type A 13 22 . In our study we could confirm the high sensitivity reported in all the studies analyzing the CADe systems. On the other hand, detection Type B failed to detect one polyp; however, the number of FPs was significantly reduced, leading to high specificity.

In the previous study published by our group 23 , EndoMind had a significantly higher number of FPs and a significantly lower FDT, compared to performance in our current work 23 . One reason might be that the EndoMind hardware that was used in the present study infers from every third frame rather than from every single frame, to reduce the number of FPs and to have less delay in the image-processing pipeline. In contrast, in our previous work the EndoMind neuronal network analyzed every single frame.

This study has some limitations. The Endo-AID system uses the EVIS X1 CV-1500 videoprocessor, therefore use of the Serie 1500 videocolonoscopes would have been a desirable option. However, the CF-HQ190 videocolonoscope has the great advantage of being supported by all the CADe systems compared in this study, including Endo-AID. Hence we excluded from our dataset all videos recorded with CF-H180 videocolonoscopes. Bearing in mind that this was a retrospective study, not all polyps detected by the endoscopist were resected and therefore the histology is not available in some cases. Finally, while our study provides valuable insights into the performance trends of different CADe systems, the retrospective and exploratory nature of the analysis limits the comparison.

In summary, our present study describes for the first time the performance of three AI polyp detection systems in the same dataset. In addition, the frame-by-frame analysis gives much more robust results and a clearer picture of how the systems perform in real conditions. It has been observed that the GI Genius software update significantly increases sensitivity. The impact on the behavior of the Endo-AID system depending on the detection type chosen has also been observed. Finally, EndoMind, a freely available system developed in a public hospital, has been shown to perform similarly to commercially available systems. Based on the outcomes presented here, clinicians might have more information to help decide which CADe system best suits their needs by selecting the one with the preferred sensitivity–specificity balance.

## Supplementary material

Supplementary material Zusatzmaterial
