# Supplementary material for: Direct comparison of multiple computer-aided polyp detection systems
Source: Endoscopy. 2023 Oct 5;56(1):63–9. doi: 10.1055/a-2147-0571 (PMC10736101; doi:10.1055/a-2147-0571)
Supplement: Supplementary file 2 — Supplementary material [file 22948supmat_10-1055-a-2147-0571.pdf]

Supplementary material

Direct comparison of multiple computer-aided polyp detection systems

Authors: Joel Troya, Boban Sudarevic, Adrian Krenzer, Michael Banck, Markus Brand, Benjamin Walter, Frank Puppe, Wolfram G. Zoller, Alexander Meining, Alexander Hann

Study flow diagram

Fig. 1s shows the study flow diagram of the colonoscopy videos included and excluded in the study. Exclusion criteria included inflammatory bowel disease, bleeding, poor bowel preparation, incomplete colonoscopy, stenosis, defective videos, serrated polyposis syndrome, endoscopic submucosal dissection clip, hemicolectomy, full-thickness resection in the rectum, lymphomas or tumors, xanthomatosis, Kaposi sarcoma and endoscopic mucosal resection.

All the colonoscopies were performed with CF-HQ190AL colonoscopes (Olympus Corporation, Japan). A standard split-dose regimen was used to prepare all the patients for the colonoscopy.

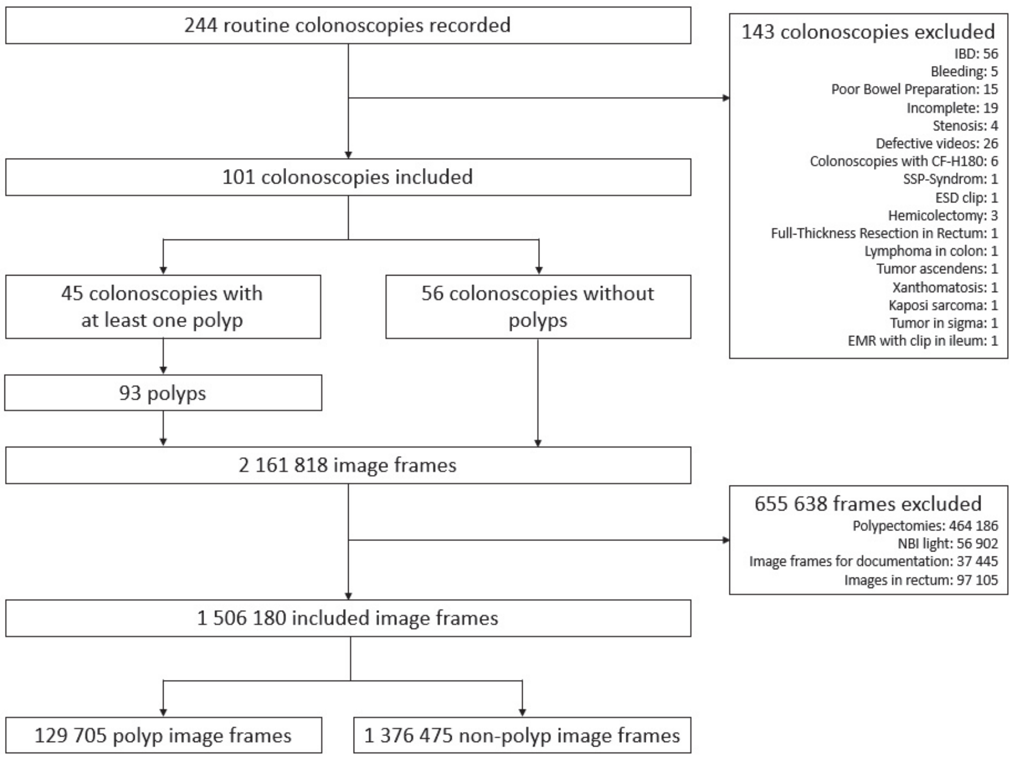

Fig. 1s Flow diagram of data selection. IBD, inflammatory bowel disease; SSP, serrated polyposis syndrome; ESD, endoscopic submucosal dissection; EMR, endoscopic mucosal resection; NBI, narrow-band imaging.

Supplementary material

Annotation methodology

A custom-made annotation tool was used to annotate in a deep frame-by-frame process all the included colonoscopies. A board-certified gastroenterologist was in charge of this process. The annotated labels included all the frames with polyps and the establishing of the withdrawal and resection times. In addition, the size and morphology of the detected polyps were annotated at this point. To exclude the presence of multiple hyperplastic polyps in the rectum, this region was excluded from the analysis. Subsequently in this study, two trained medical students drew bounding boxes in each of the frames that contained a polyp to obtain its precise location in the image and setting the ground-truth. All these bounding boxes were then checked an additional time by a second board-certified gastroenterologist and corrections were manually performed if necessary. In addition, the hole annotation process was supervised by an experienced biomedical engineer.

To perform the annotations and revisions of the frames, a custom-software revision tool was developed using the library PyQt5 in Python 3.8 (**Fig. 2s**). The revision tool has a main screen where the image is displayed. The tasks or images to review are listed in a scroll tab in the right of the screen. The ground-truth bounding boxes previously annotated are displayed in red and can be reset and corrected. The speed of image visualization could be chosen with a slider. If the polyp was not visible in the screen, the endoscopist could press the button “Where is the polyp?” which would generate the label “Polyp not identified” in the dataset. False positive detections were also screened to discard the presence of additional lesions. In comparison to the previously published analysis [1], we included only white light images since the Olympus EndoAID predicts only in this mode. We also excluded all endoscopies performed with a colonoscope CF-H180 since Endo-AID does not support this endoscope type.

Supplementary material

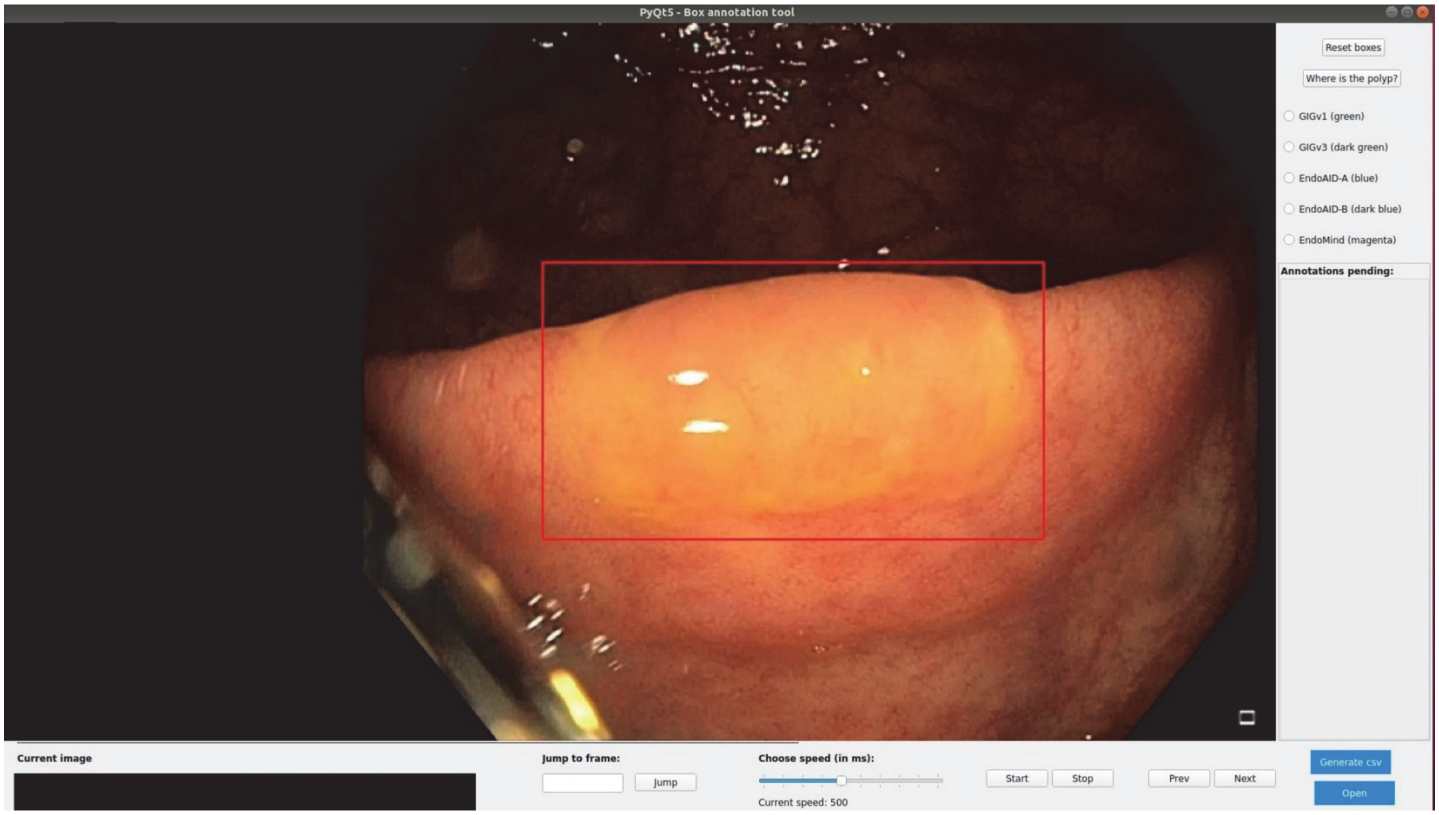

Fig. 2s Screenshot of the custom review program developed to check the ground-truth annotations.

Supplementary material

Bounding box detection and localization

In order to detect the presence of bounding boxes displayed by a computer-aided polyp detection (CADe) system in an image frame, two different approaches were used. On the one hand, classical image transformation methods allowed us to detect and locate the bounding boxes. On the other hand, a neural network trained to detect the bounding boxes was developed. Both algorithms ran in parallel. When there were discrepancies between them, the user was deciding if there was presence of bounding box or not and the location. With this methodology, we ensure that all the bounding boxes were correctly determined.

The following small sections show the preprocessing and detection algorithms used on the "classical" approach and details about the developed neural network trained to detect the bounding boxes.

Preprocessing

```
import numpy as np
import cv2
from skimage import motphology
from skimage.morphology import square

# Crop the image
image = image[:, 320:1410, :]

# Set minimum and maximum HSV value thresholds
lower = np.array([22, 144, 95])
upper = np.arra([62, 246, 255])

# Create HSV image and create a mask using the thresholds
hsv = cv2.cvtColor(image, cv2.COLOR_BGR2HSV)
mask = cv2.inRange(hsv, lower, upper)

# Slice the hsv
imask = mask>0
image = np.zeros_like(hsv, np.uint8)
image[imask] = hsv[imask]

# Separate by channel
b, image, r = cv2.split(image)

# Blur the image
image = cv2.bilateralFilter(image, 9, 25, 25)

# Sharpen the image
kernel = np.array([[0, -1, 0], [-1, 5, -1], [0, -1, 0]])
image = cv2.filter2D(src=blurred, ddepth=-1, kernel=kernel)

# Apply dilation
image = morphology.dilation(image, square(3))
```

Supplementary material

```
# Apply binary threshold
(thresh, image) = cv2.threshold(res, 128, 255, cv2.THRESH_BINARY | cv2.THRESH_OTSU)

# Apply 3x dilation
for counter in range(3):
    image = morphology.dilation(image, square(3))

return image
```

Detecting boxes

```
import cv2
import numpy as np

#Find contours
contours, hierarchy = cv2. findContours(image, cv2.RETR_TREE, cv2.CHAIN_APPROX_SIMPLE)

# If the contour area is bigger than a threshold, then it is probably a bounding box
bboxes =[]
for contour in contours:
    if cv2.contourArea(contour) > 800:
        bboxes.append(cv2.boundingRect(contour))

# Check if the bounding boxes are inside bounding boxes
bboxes = is_inside(bboxes)

# Post-process boxes using intersections, overlapping and location in the image
bboxes = process_coordinates(bboxes)
```

Artificial intelligence to detect boxes

For model training we used the pretrained ResNet50 based on deep learning and provided by the *tensorflow.keras.applications* library. The model was trained with 6575 positive bounding-box images and 6314 negative bounding-box images in a batch size of 29 for 5 epochs. Binary crossentropy from the *keras* library was used as a loss function and a learning rate of 0.0001 was set for the Adam optimizer. The model was trained with a NVIDIA RTX3080 graphic processing unit.

Supplementary material

Secondary outcomes

The secondary outcomes analyzed in this study are: the intersection over the union (IoU), the first detection time (FDT) and the false-positive rate. All of them have been analyzed in a frame-by-frame manner.

The IoU is a standard metric used to measure the accuracy of object detection by comparing the overlap between a predicted bounding box and a ground-truth bounding box with the total area of both. The IoU was calculated for each displayed bounding box of each CADe system and was used to discriminate between correct and incorrect bounding boxes and calculate each CADe system's sensitivity **(Fig. 3s)**. A bounding box with IoU>0, was regarded as correct (TP). This is equivalent to considering the activation correct if there is an intersection between the gold-standard and the predicted bounding box. As analyzed by Tran et al., this approach might better approximate the clinical need [2]. The FDT was another secondary outcome. FDT is defined as the minimum time required for the CADe to detect a polyp, without considering the latency of the system. Lastly, the FP rate was analyzed as the proportion between the number of non-polyp containing images in which the CADe falsely predicted a polyp and the total number of frames.

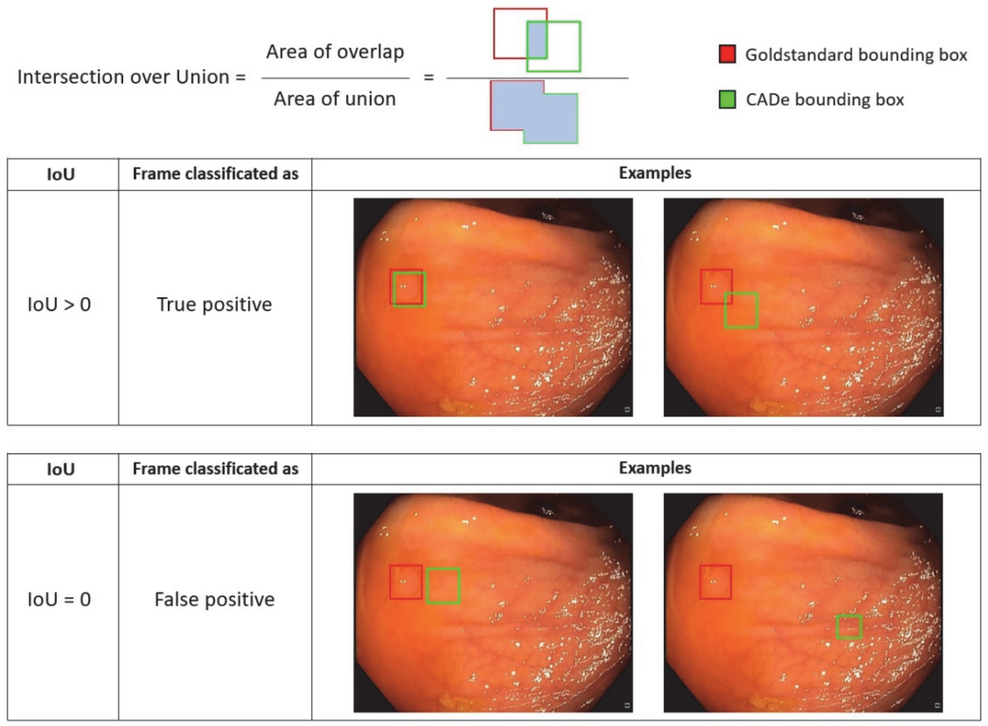

Fig. 3s Explanation of the intersection over union metric and the criteria used to classify the polyp-containing image frames into true or false according to the intersection over union value.

Supplementary material

Statistical analyses

To test if the data follows a Gaussian distribution, the Shapiro-Wilk test was used. To test if there are significant differences in the per-polyp sensitivity and in the FDT distributions across the different CADe systems, the Mann-Whitney U test was used. To test if the mean values of the IoU distributions were significantly different, the t-test was used. The point estimates for the mean per-frame sensitivity, the mean FDT, and the mean IoU are presented with a 95% confidence interval. The point estimates for the median per-frame sensitivity for polyp morphology are presented with the interquartile range. All statistics were performed using SciPy package in Python version 3.8 [3].

Ethics

The retrospective analysis of data was reviewed and approved by the Ethics Committee of the University Hospital Würzburg, approval number 2021032901. Patients were not required to give informed consent for this retrospective analysis.

References

1. Brand M, Troya J, Krenzer A et al. Frame-by-Frame Analysis of a Commercially Available Artificial Intelligence Polyp Detection System in Full-Length Colonoscopies. Digestion 2022; 103: 378-385. doi:10.1159/000525345
2. Thuy Nuong Tran, Tim Adler, Amine Yamlahi et al. Sources of performance variability in deep learning-based polyp detection. arXiv:221109708 2022. doi:https://doi.org/10.48550/arxiv.2211.09708
3. Virtanen P, Gommers R, Oliphant TE et al. SciPy 1.0: fundamental algorithms for scientific computing in Python. Nat Methods 2020; 17: 261-272. doi:10.1038/s41592-019-0686-2

Thieme

Supplementary material

Table 1s. Characteristics of the patients and the polyps included in the dataset.

Characteristic		Value
**Sex**	Male, n (%)	47 (46.53)
	Female, n (%)	54 (53.47)
**Age, mean (range)**		60 (19-89)
**BBPS, mean (range)**		7.45 (6-9)
**Colonoscopy purpose**	Screening, n(%)	27 (26.73)
	Symptomatic, n(%)	74 (73.27)
**Polyps, n**		93
**Polyps per patient, mean (range)**		0.92 (0-6)
**Polyp detection rate, (%)**		44.55
**Adenoma detection rate, (%)**		32.67
**Histology, n (%)**	Adenoma	39 (41.94)
	Hyperplastic	13 (13.98)
	SSA	17 (18.28)
	Other	24 (25.81)
**Paris classification, n (%)**	0-Ip	5 (5.38)
	0-Is	28 (30.11)
	0-IIa	60 (64.52)
**Size, n (%)**	< 5 mm	54 (58.06)
	5 – 10 mm	24 (25.81)
	> 10 mm	15 (16.13)
**Location, n (%)**	Right colon	50 (53.76)
	Left colon	28 (30.11)
	Rectum	15 (16.13)

SSA, Sessile serrated adenoma

Supplementary material

Table 2s First detection time (FDT) for each CADe system analyzed.

	First detection time, **mean** (95% Confidence Interval), ms													
	Overall	Paris Classification			Size			Location			Histology			
		0-Ip	0-Is	0-IIa	<5 mm	5-10 mm	>10 mm	Right colon	Left colon	Rectum	HP (16)	SSL (18)	Adenoma (43)	Other (31)
**GI Genius version 1**	**1510** (1125-1895)	**520** (216-824)	**913** (493-1333)	**1872** (1325-2419)	**1475** (1002-1949)	**1214** (610-1817)	**2111** (567-3654)	**1913** (1283-2542)	**1032** (522-1542)	**1062** (524-1601)	**1254** (338-2170)	**1639** (847-2432)	**1262** (784-1741)	**1961** (820-3102)
**GI Genius version 2**	**607** (411-803)	**453** (0-1148)	**571** (268-875)	**637** (366-909)	**643** (357-929)	**689** (297-1081)	**329** (124-533)	**630** (374-886)	**729** (255-1202)	**307** (125-488)	**585** (74-1096)	**692** (137-1246)	**663** (376-951)	**472** (22-922)
**EndoAID Type A**	**659** (410-909)	**387** (0-813)	**287** (141-433)	**856** (484-1229)	**589** (274-904)	**518** (153-883)	**1140** (167-2113)	**784** (399-1169)	**550** (122-978)	**449** (0-908)	**713** (73-1353)	**790** (254–1326)	**408** (124-691)	**947** (200-1695)
**EndoAID Type B**	**1316** (951-1682)	**960** (0-2650)	**699** (314-1083)	**1640** (1121-2158)	**1101** (675-1526)	**1360** (724-1995)	**2009** (534-3484)	**1484** (934-2033)	**1142** (488-1795)	**1096** (333-1858)	**1274** (491–2057)	**2071** (1099-3042)	**744** (373-1114)	**1754** (690-2818)
**EndoMind**	**1083** (627-1539)	**167** (0-340)	**376** (193-559)	**1489** (808-2171)	**1007** (488-1525)	**1001** (97-1906)	**1489** (0-3323)	**1234** (552-1916)	**715** (142-1289)	**1267** (0-2757)	**856** (0-1853)	**1392** (97-2687)	**808** (219-1397)	**1435** (233-2636)

ms: milliseconds; HP: Hyperplastic polyp; SSL: Sessile serrated lesion
